# When size matters: advantages of weighted effect coding in observational studies

**DOI:** 10.1007/s00038-016-0901-1

**Published:** 2016-10-28

**Authors:** Manfred te Grotenhuis, Ben Pelzer, Rob Eisinga, Rense Nieuwenhuis, Alexander Schmidt-Catran, Ruben Konig

**Affiliations:** 1 Radboud University, Nijmegen, The Netherlands; 2 Stockholm University, Stockholm, Sweden; 3 University of Cologne, Cologne, Germany

## Introduction

To include nominal and ordinal variables as predictors in regression models, their categories first have to be transformed into so-called ‘dummy variables’. There are many transformations available, and popular is ‘dummy coding’ in which the estimates represent deviations from a preselected ‘reference category’. A way to avoid choosing a reference category is effect coding, where the resulting estimates are deviations from a grand (unweighted) mean. An alternative for effect coding was given by Sweeney and Ulveling in 1972, which provides estimates representing deviations from the sample mean and is especially useful when the data are unbalanced (i.e., categories holding different numbers of observation). Despite its elegancy, this weighted effect coding has been cited only 35 times in the past 40 years, according to Google Scholar citations (more recent references include Hirschberg and Lye 2001 and Gober and Freeman 2005). Furthermore, it did not become a standard option in statistical packages such as SPSS and R. The aim of this paper is to revive weighted effect coding illustrated by recent research on the body mass index (BMI) and to provide easy-to-use syntax for SPSS, R, and Stata on http://www.ru.nl/sociology/mt/wec/downloads. For didactical reasons we apply OLS regression models, but it will be shown that weighted effect coding can be used in any generalized linear model.

One favored way of transforming categories into dummy variables is dummy coding (Hardy 1993). In this transformation, units (e.g., respondents) within a specific category are coded as 1 and all other units as 0 on a new (dummy) variable. The parameter for this dummy variable then is the estimated mean difference in the scores on the dependent variable between that specific category and the chosen reference category. As an example we examine the well-known relationship between BMI and Educational attainment (Hermann et al. 2011), a categorical variable containing three levels: low, middle, and high and we use the first level (low) as the point of reference (see Table 1 for the coding scheme). In Table 2 the empirical results are shown, using random cross-sectional samples (total *n* = 3314) drawn from the general Dutch population aged 18–70, in 2000, 2005, and 2011 including self-reported body length and weight (Eisinga et al. 2002, 2012a, b). In Model 1 of Table 2, second column, an estimated mean BMI of 26.15 is found for the intercept, and represents the estimated mean BMI for the ‘low’ (reference) category. Respondents with mid-level education have an estimated mean BMI that is 1.17 points lower and for the high educated respondents the estimated mean BMI lies 1.85 points lower, both compared to the respondents with low levels of education. These deviations from the mean BMI in the low educated differ significantly from 0. To test whether the difference of 0.68 BMI points (1.85–1.17) between the middle and high educated respondents is significant, one has to change the reference category (this difference turned out to be significant as well, details can be found on the website that goes with this text).

**Table 1:** Coding schemes for dummy coding, effect coding, and weighted effect coding (example with 3 levels of educational attainment and lowest educational level omitted from the regression model)

Dummy variables	Dummy coding		Effect coding		Weighted effect coding	
	Middle_dc_	High_dc_	Middle_ec_	High_ec_	Middle_wec_	High_wec_
Categories						
Low	0	0	−1	−1	−(*n*_m_/*n*_l_)^a^	−(*n*_h_/*n*_l_)^b^
Middle	1	0	1	0	1	0
High	0	1	0	1	0	1

^a^ *n*_m_ = number of observations (*n*) in category Middle, *n*_l_ = *n* in category Low

^b^ *n*_h_ = *n* in category High

**Table 2:** Ordinary least squares (OLS) regression effects on the body mass index (BMI), using dummy coding, effect coding, and weighted effect coding without controls (Model 1) and with controls (Model 2), number of cases per category between brackets (*n*) Data source: (Eisinga et al. 2000, 2012a, b), total *n* = 3314

OLS effects on BMI	Dummy coding		Effect coding		Weighted effect coding	
	*b*-estimates	*t* values	*b*-estimates	*t* values	*b*-estimates	*t* values
**Model 1**						
Intercept	26.15	184.15	25.14	368.75	24.98	383.32
Education						
Low (698)	0.00 (ref)		1.00	9.44	1.17	9.27
Middle (1419)	−1.17	−6.74	−0.16	−1.82	−0.00 ns	−0.00
High (1197)	−1.85	−10.36	−0.84	−9.12	−0.68	−7.87
Variance explained	3.1%		3.1%		3.1%	
**Model 2**						
Intercept	25.88	143.74	25.10	373.04	24.98	394.00
Education						
Low (698)	0.00 (ref)		0.74	6.98	0.85	6.78
Middle (1419)	−0.73	−4.22	0.01 ns	0.15	0.12	1.67
High (1197)	−1.49	−8.45	−0.75	−8.31	−0.64	−7.59
Control variables						
Sex						
Male (1561)	0.00 (ref)		0.24	3.78	0.26	3.78
Female (1753)	−0.48	−3.78	−0.24	−3.78	−0.23	−3.78
Age (log)^a^	2.42	12.90	2.42	12.90	2.42	12.90
Year of interview						
2000 (987)	0.00 (ref)		−0.20	−2.19	−0.20	−2.08
2005 (1351)	0.20	1.31	−0.00 ns	−0.04	−0.00 ns	−0.03
2010 (937)	0.41	2.48	0.21	2.22	0.21	2.11
Variance explained	8.4%		8.4%		8.4%	

*ns* not significant (*t* value <1.65), *t* values are presented for illustrative purposes

^a^Because the relationship between age and BMI turned out to be positive and non-linear, we used the natural logarithm of age and mean centered log(age) to ensure that the intercept equals the sample mean of 24.98 in weighted effect coding

After controlling for sex (also dummy coded), (log)age, and year of interview (dummy coded) (these three control variables were obtained from Krul et al. 2011; Hermann et al. 2011; Stevens et al. 2012), the initial differences of the middle and high educated with the low (reference) category are somewhat smaller (see Table 2, Model 2). The controlled estimates of the dummy variables, however, still represent the estimated mean difference between a specific category (here: middle and high) and the reference category (low).

### Effect coding

Effect coding (also known as deviation contrast, or ANOVA coding) was developed out of the desire to test all category means against one overall mean value (Hardy 1993). By doing so one avoids preselecting a (frequently arbitrary) reference category as in dummy coding. In general terms, effect coding uses a constraint in which the sum of all estimates (*b*) in a set of dummy variables (*I*) is 0:

1 $$\mathop \sum \limits_{i = 1}^{I} b_{i} = 0.$$

As a consequence, parameters related to effect coded dummy variables are deviations from an unweighted grand mean. This grand mean is the average of the estimated means of all categories of a specific variable, *without* taking into account the possible unequal number of observations per category (see also Table 1 for the coding scheme). In our example, the effect coded dummy variables for education show that the lower educated respondents differ most from the grand mean of 25.14 (intercept), namely 1 BMI point (see Table 2, Model 1). The middle-educated are on average 0.16 points below that grand mean and in high educated individuals the difference is −0.84. Note that the predicted mean BMI is 26.14 (25.14 + 1) for the low category, 24.98 (25.14–0.16) for the middle category, and 24.3 (25.14–0.84) for the high category. The grand mean of 25.14 is the average of these three BMI means ((26.14 + 24.94 + 24.3)/3). So in effect coding, the reference, i.e., the grand mean to which the statistical tests relate, does not depend on possible different numbers of observations per category. After taking into account the three aforementioned control variables, the deviations from the grand mean (which has shifted somewhat to 25.1) become smaller within all three categories of education and for the middle-educated the deviation approaches 0 (see Table 2, Model 2).

Effect coding is well-suited whenever the data are balanced, i.e., when the numbers per category of a nominal or ordinal variable are (roughly) equal. We like to note that this is not a necessary condition for the sample data; it suffices to assume a population with such a balanced design. The usefulness of effect coding is illustrated in Table 2, Model 2, where effect coded estimates for males and females are exact counterparts (0.24 vs. −0.24), which is congruent with the (almost) 1:1 sex-ratio in the target population.

The effects for the years 2000, 2005, and 2011 are also non-problematic, because population sizes did not change much over these years. However, the number of individuals differs profoundly across the three main educational categories in the Dutch population and this is also reflected in our sample (see Table 2 for the numbers of observation per category). If a researcher wants to take into account these different sizes, effect coding is less appropriate as we will show in the next paragraph.

### Weighted effect coding

To take into account the unequal number of observations across categories, Sweeney and Ulveling (1972) introduced a coding scheme that enables testing against the sample (arithmetic) mean. To achieve this, the sum of all weighted (*w*_*i*_) estimates (*b*) in a set of dummy variables (*I*) equals 0:

2 $$\mathop \sum \limits_{i = 1}^{I} w_{i} b_{i} = 0.$$

The weight (*w*_*i*_) in Eq. 2 equals −(*n*_*x*_/*n*_o_), where *n*_*x*_ stands for the number of observations in category *x* and *n*_o_ is the number of observations in category o. The latter category is omitted from the regression model as it is statistically redundant. As a result of weighting, the midpoint or reference shifts away from the unweighted grand mean to the weighted sample mean. The procedure is, therefore, known as weighted effect coding. Note that contrary to the weights *w*_*i*_ in Eq. 2, the weights in Eq. 1 in fact all are set to 1 (and, therefore, omitted from Eq. 1), ignoring the possible unequal number of observations across categories (see Table 1 for the coding differences between weighted effect coding and effect coding).

In our sample, we have 698 respondents who are low educated, 1419 are middle educated and there are 1197 high educated respondents. Following Sweeney and Ulvering, we created the weighted effect coded (wec) dummy variable middle_wec_ with code 1 for respondents in the middle category and code 0 for all high educated respondents. The code or weight *w*_*i*_ for respondents in the omitted low educated category equals −1419/698 in the dummy variable middle_wec_ (see also Table 1). For the dummy variable high_wec_ the codings are: 1 for high, 0 for middle, and −1197/698 (*w*_*i*_) for low. Note that the denominator in *w*_*i*_ equals the number of observations (698) in the omitted category (low). With these codings, we can estimate the parameters for the middle and high educated. To have an estimate for the low educated as well, we excluded the category high from the regression model and included the dummy low_wec_ (coded 1 for low educated, 0 for middle educated and −698/1197 (*w*_*i*_) for high educated) and the dummy middle_wec_ (coded 1 (middle), 0 (low), and −1419/1197 (high)), details can be found on our website.

According to the results in Model 1 of Table 2, the low educated have an estimated mean BMI that is 1.17 points higher than the actual sample mean of 24.98. The middle category does not differ significantly from the sample mean, whereas the high educated respondents’ mean BMI lies 0.68 points lower. If we compare these results with effect coding then the different outcomes for the middle educated are most clear. The reason for this lies in the shifted reference: the estimated BMI for the middle educated equals 24.98, so the difference with the sample mean, which also happens to be 24.98, is 0. However, when compared with the grand mean of 25.14 the effect coded estimate for middle educated is −0.16. The latter outcome is of much less interest than the former, because the observed differences in the number of observations between the three education categories are considered relevant as they reflect important size differences in the population.

We again expanded the model by including the control variables sex (weighted effect coded), year of interview (weighted effect coded), and (log)age (see Table 2, Model 2). As a result of controlling, especially the deviation of the low education category changed (from +1.17 to +0.85). Note that because the intercept represents the sample mean, it retains the same value (24.98), whether control variables are included (Model 2) or not (Model 1). Again the difference in estimates for the middle educated is most striking: in weighted effect coding the difference with the sample mean is 0.12 and significant, whereas in effect coding the difference with the grand mean is almost 0. In other words, the estimated mean BMI among the middle educated respondents is almost identical to the grand mean after controlling for age, sex, and year of interview, but lies 0.12 above the sample mean. Because the numbers per category of education differ in the Dutch population, the latter is a more informative and realistic outcome. Note that for the variables sex and year of interview it is rather irrelevant whether effect coding or weighted effect coding is being used. Further note that in Table 2 the explained variances are equal in all three models 1 and in all three models 2. The only difference is the point of reference. In dummy coding this reference relates to a specific, existing category, in effect coding it is a grand mean (neglecting the possible unbalance in the data), while in weighted effect coding the point of reference is the sample mean.

In general, the results from effect coding and weighted effect coding increasingly deviate as the differences between the numbers of observation per category increase. For instance, for the following category means 2, 3, and 10, effect coding uses 5 ((2 + 3 + 10)/3) as a reference, whereas in weighted effect coding this is rather close to 10 if the bulk of observations (say 80%) is located in the category with mean = 10 (example: 2 × 0.1 + 3 × 0.1 + 10 × 0.8 = 8.5).

In sum, weighted effect coding is preferred over effect coding if a categorical variable has categories of different sizes, and if these differences are considered relevant. Contrary to most experimental designs in which the data are often balanced, this relevancy is often apparent when cross-sectional surveys (or other observational data) are being analyzed.

### Weighted effect coding in generalized linear models

In the previous section we showed that in weighted effect coding, sets of dummies are tested against the sample mean of the dependent variable Y. Of course it depends upon the scaling of Y to what this sample mean relates. If one uses a variable like BMI in an OLS regression model, then this mean is the observed sample (arithmetic) mean in BMI across all respondents in the model. If one would log-transform BMI scores first, for instance because the original BMI distribution is highly skewed, then the sample mean is the average of all log-transformed BMI scores. Likewise, if a researcher wishes to investigate obesity (BMI > 30) and, therefore, uses a dichotomy of BMI in a logistic regression analysis, then the mean to be tested against is the average of all log odds. We provide some examples of weighted effect coding with log-transformed and dichotomous variables on our website.

### Weighted effect coding in SPSS, Stata, and R

Weighted effect coding has not yet been included in the popular statistical packages R and SPSS. Therefore, we designed for these statistical packages easy-to-use syntax. In Stata there is only the possibility to obtain the weighted effect estimates using the post-estimation command ‘contrast’. We, therefore, wrote a Stata ado-file to create the weighted effect dummies before using any regression model. All syntax, example data on BMI, and outcomes can be downloaded from our website. In a follow-up *Hints* and *Kinks* we discuss novel interactions between weighted effect coded dummy variables representing the additional effects over and above the main effects obtained from a model without these interactions.
